# Exosomes containing differential expression of microRNA and mRNA in osteosarcoma that can predict response to chemotherapy

**DOI:** 10.18632/oncotarget.18373

**Published:** 2017-06-06

**Authors:** Ji-Feng Xu, Ya-Ping Wang, Shui-Jun Zhang, Yu Chen, Hai-Feng Gu, Xiao-Fan Dou, Bing Xia, Qing Bi, Shun-Wu Fan

**Affiliations:** ^1^ Department of Cardiology, 2nd Affiliated Hospital, School of Medicine, Zhejiang University, Zhejiang 310009, P.R. China; ^2^ Department of Orthopedics, Zhejiang Provincial People's Hospital, Hangzhou Medical College, Hangzhou, Zhejiang 310014, P.R. China; ^3^ Department of Orthopedics, Sir Run Run Shaw Hospital, School of Medicine, Zhejiang University, Hangzhou, Zhejiang 310016, P.R. China

**Keywords:** osteosarcoma (OS), chemotherapy sensitivity, exosome, microRNA, poor chemotherapeutic response

## Abstract

A major challenge in osteosarcoma (OS) is the selection of the most effective chemotherapeutic agents for individual patients, while the administration of ineffective chemotherapy increases mortality and decreases quality of life in patients. This emphasizes the need to evaluate every patient's probability of responding to each chemotherapeutic agent. We developed a profiling strategy for serum exosomal microRNAs and mRNAs in OS patients with differential chemotherapeutic responses. Twelve miRNAs were up regulated and 18 miRNAs were under regulated significantly in OS patient with poor chemotherapeutic response when compared with those in good chemotherapeutic response (p<0.05). In addition, miR-124, miR133a, miR-199a-3p, and miR-385 were validated and significantly reduced in poorly responded patients with an independent OS cohort. While miR-135b, miR-148a, miR-27a, and miR-9 were significantly over expressed in serum exosomes. Bioinformatic analysis by DIANA-mirPath demonstrated that Proteoglycans in cancer, Hippo signaling pathway, Pathways in cancer, Transcriptional misregulation in cancer, PI3K-Akt signaling pathway, Ras signaling pathway, Ubiquitin mediated proteolysis, Choline metabolism in cancer were the most prominent pathways enriched in quantiles with the miRNA patterns related to poor chemotherapeutic response. Messenger RNAs(mRNAs) includingAnnexin2, Smad2, Methylthioadenosine phosphorylase (MTAP), Cdc42-interacting protein 4 (CIP4), Pigment Epithelium-Derived Factor (PEDF), WW domain-containing oxidoreductase (WWOX), Cell division cycle 5-like (Cdc5L), P27 were differentially expressed in exosomes in OS patients with different chemotherapeutic response. These data demonstrated that exosomal RNA molecules are reliable biomarkers in classifying osteosarcoma with different chemotherapy sensitivity.

## INTRODUCTION

Osteosarcoma (OS) is one of the most common bone malignancy, which predominately affects young people. The current therapy is aimed to inhibit tumor growth and metastasis by chemotherapy together with clinical surgery, while the 5-year survival rate is still poor [1, 2]. However, chemo-resistance and recurrence are two major challenges in OS facing with physicians. Tumor necrosis, which is evaluated at the time of resection following chemotherapy, is considered as an important prognostic indicator in OS. Unfortunately, less than 30% of patients are resistant to the chemotherapeutic agents. Chemotherapy inability to cure metastatic disease is possibly responsible for OS progression. It is recently demonstrated that patients with ≥90% tumor necrosis (good response) will have more favorable prognosis similar to the preoperative regimen [3]. However patients with <90% tumor necrosis (poor response) will have a higher risk of relapse and poor outcome. Thus, it is still hard to identify at the time of initial diagnosis the patients who are likely to have a poor response to chemo-therapy. Reliable biomarkers are required for diagnosis of patients with poor response, and tracking the disease progression of osteosarcoma poor outcome.

MicroRNAs (miRNAs) are small (22-nt) endogenous noncoding RNAs that can bind to the 3’UTR of target mRNAs to mediate translation or degradation [4]. Disease-associated changes in miRNA expression in OS samples were determined recently [5, 6]. Exosomes, which carry the transferred miRNAs, are considered as novel regulators of cellular function [7–9]. MiRNAs transfer can also cause the physiological changes in their recipient cells, which was demonstrated by miRNAs moving from cancer cells to endothelial cells, promoting tumor metastasis [7–9]. In addition, cancer cells can also receive exosomal miRNAs secreted from immune cells, exerting as anti-proliferative effect on the tumor cells [10]. However, the importance of exosomes in the pathogenesis of osteosarcoma response to chemotherapy has yet been established. Further, alterations of exosomal miRNA or mRNA content in sera from OS patients with differential response to chemotherapy have not yet been described. The primary goal of this study was to develop a predictive model to classify OS in response to preoperative chemotherapy using exosomal microRNA expression profiling, and to explore their potential as biomarkers in OS patients who has poor chemotherapeutic response.

## RESULTS

### MiRNAs were differentially expressed in sera exosomes in OS patients with different response to chemotherapy

**Figure 1:**
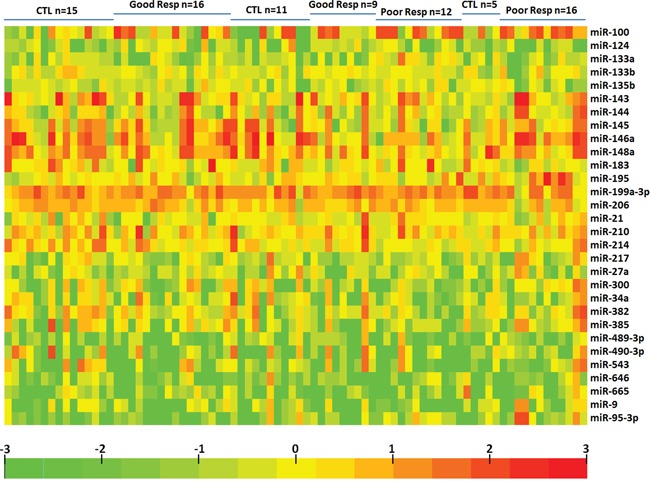
Heatmap of exosomal differential miRNA profiles in osteosarcoma patients with different chemotherapeutic responses Heatmap representation of the mean fold change in differential miRNA signature. Two-dimensional grid matrix displaying 30exosomal miRNAs was obtained by the functional heat-map in R. Columns refer to time course comparison: 31 healthy controls, 25good response and 28poor response. Rows stand for the 30 differential miRNAs. Each entry of the grid refers to relative fold (log2) between the expression level of a given miRNA in exosome relative to U6 in healthy controls. The color of each entry is determined by the value of that fold difference, ranging from green (negative values) to red (positive values).

**Figure 2:**
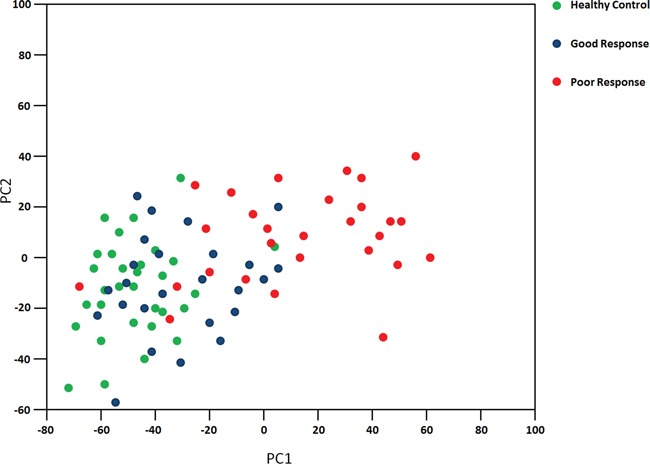
Principal component analysis The plots for disease phenotypes (healthy, good chemotherapeutic response and poor chemotherapeutic response) were performed as principal component analysis among all samples based on miRNA profiles.

The characteristics and structures of exosomes isolated from serum in this study were confirmed by FACS analysis of surface CD63 and electron microscopy performed as previously described [11]. To identify differentially expressed exosomal microRNA pattern in OS patients with good and poor chemotherapeutic responses, we profiled the expression of 746 miRNAs by using TaqMan miRNA arrays in a pilot cohort of 31 healthy controls, 25 OS patients with good response and 28 OS patients with poor response (Supplementary Table 1). The relative abundances of the detected exosomic miRNAs were normalized in each sample to U6. The data indicated that 164 miRNAs (22%) could be detected (assays giving Ct < 34, miRNA was classed as detectable). Thirty exosomic miRNAs were differentially expressed in OS patients with poor chemotherapeutic response compared to those with good response. Among them, we found that 12exosomal miRNAs were up regulated and 18 miRNAs were under regulated significantly (p<0.05) in exosomes from OS patients with poor chemotherapeutic response when compared those with good chemotherapeutic response (Figure 1 and Table 1). In addition, the plots for disease phenotypes (healthy, good chemotherapeutic response and poor chemotherapeutic response) were performed as principal component analysis (PCA) among all samples based on miRNA profiles (Figure 2). Healthy controls and good chemotherapeutic response were not correlated with the first and second principal components. Patients with poor chemotherapeutic response was correlated with the first PC (p<0.01), which suggested that the statistical results from differential miRNA expression profiling would be affected by principal components when testing differential exosomal miRNAs expression. Taken all together, these data indicated that exosomal miRNAs were with clinical significance in prediction in OS patients with poor response to chemotherapy.

**Table 1:** Differential miRNA expression in exosomes between differentresponse to chemotherapy in osteosarcoma

Good Responders vs. Control			Poor Responders vs. Contro		
miR-Name	fold change	Adjusted p-value	miR-Name	fold change	Adjusted p-value
miR-21	0.824	0.0516	miR-21	11.392	0.0001
miR-27a	0.914	0.1021	miR-27a	6.774	0.0104
miR-148a	1.257	0.1875	miR-148a	5.540	0.0084
miR-135b	1.181	0.0533	miR-135b	4.347	0.0003
miR-9	1.125	0.2152	miR-9	2.676	0.0325
miR-214	1.165	0.0567	miR-214	2.621	0.0063
miR-210	0.959	0.0797	miR-210	2.428	0.0005
miR-300	2.028	0.0730	miR-300	2.173	0.0035
miR-665	1.705	0.0534	miR-665	1.778	0.0034
miR-145	2.329	0.0880	miR-145	1.729	0.0059
miR-543	0.940	0.2766	miR-543	1.516	0.0033
miR-382	0.946	0.0663	miR-382	1.424	0.0038
miR-183	1.257	0.2041	miR-183	0.829	0.0030
miR-133b	1.014	0.0640	miR-133b	0.774	0.0124
miR-34a	0.914	0.2928	miR-34a	0.768	0.0144
miR-490-3p	1.079	0.2925	miR-490-3p	0.753	0.0089
miR-646	0.646	0.2652	miR-646	0.747	0.0026
miR-146a	0.737	0.0609	miR-146a	0.722	0.0046
miR-144	0.763	0.2197	miR-144	0.678	0.0063
miR-217	1.035	0.0672	miR-217	0.669	0.0121
miR-489-3p	0.953	0.0601	miR-489-3p	0.655	0.0002
miR-100	1.173	0.2096	miR-100	0.616	0.0079
miR-95-3p	0.511	0.0630	miR-95-3p	0.616	0.0251
miR-143	0.678	0.1996	miR-143	0.486	0.0003
miR-195	1.035	0.0645	miR-195	0.467	0.0138
miR-206	1.853	0.0547	miR-206	0.435	0.0103
miR-133a	1.021	0.0810	miR-133a	0.423	0.0120
miR-124	1.094	0.0706	miR-124	0.403	0.0233
miR-199a-3p	1.035	0.2628	miR-199a-3p	0.240	0.0130
miR-382	1.569	0.1396	miR-382	0.028	0.0123

### Comparative pathway analyses

We further determine the biologic pathways affected by exosomal miRNAs related with poor chemotherapeutic response, DIANA-mirPath was applied on the dysregulated exosmal miRNAs. Thirty KEGG biological processes were significantly enriched (p<0.05, FDR corrected) in poor chemotherapeutic response. Among them, Proteoglycans in cancer (p=6.454E-09), Adherens junction (p=2.600E-07), ECM-receptor interaction (p=4.887E-07), Hippo signaling pathway (p=5.721E-07), TGF-beta signaling pathway (p=2.408E-06), Rap1 signaling pathway (p=2.388E-05), Pathways in cancer (p=4.904E-05), Prostate cancer (p=9.959E-05), Transcriptional misregulation in cancer (p=1.580E-04), Endocytosis (p=2.308E-04), Regulation of actin cytoskeleton (p=3.968E-04), Focal adhesion (p=5.100E-04), PI3K-Akt signaling pathway (p=1.011E-03), Ras signaling pathway (p=1.515E-03) were the most prominent pathways enriched in quantiles with differential exosomal miRNAs in poor chemotherapeutic response (Table 2), suggesting that these biologic pathways were involved in poor chemotherapeutic response. The KEGG pathway “Proteoglycans in cancer” was significantly altered in poor chemotherapeutic response with 10 miRNAs including miR-543, miR-300, and miR-217, targeting 76 genes in the pathway map (Supplementary Figure 1). The KEGG pathway “Pathways in cancer” was also significantly altered in poor chemotherapeutic response with 10 miRNAs (hsa-miR-133b, hsa-miR-199a-3p, hsa-miR-206, hsa-miR-217, hsa-miR-300, hsa-miR-489-3p, hsa-miR-490-3p, hsa-miR-543, hsa-miR-646, and hsa-miR-665) targeting 143 genes in the pathway map (Supplementary Figure 2). The KEGG pathway “Transcriptional misregulation in cancer” was significantly enriched in poor chemotherapeutic response with 10 miRNAs (hsa-miR-133b, hsa-miR-199a-3p, hsa-miR-206, hsa-miR-217, hsa-miR-300, hsa-miR-490-3p, hsa-miR-543, hsa-miR-646, hsa-miR-665, hsa-miR-95-3p) targeting 65 genes in the pathway (Supplementary Figure 3).

**Table 2:** Biologic pathways enriched by differentially expressed exosomal miRNAs

KEGG pathway	p-value
Prion diseases (hsa05020)	2.820E-09
Signaling pathways regulating pluripotency of stem cells (hsa04550)	2.820E-09
**Proteoglycans in cancer (hsa05205)**	**6.454E-09**
Adherens junction (hsa04520)	2.600E-07
ECM-receptor interaction (hsa04512)	4.887E-07
**Hippo signaling pathway (hsa04390)**	**5.721E-07**
TGF-beta signaling pathway (hsa04350)	2.408E-06
Axon guidance (hsa04360)	1.494E-05
Rap1 signaling pathway (hsa04015)	2.388E-05
**Pathways in cancer (hsa05200)**	**4.904E-05**
Prostate cancer (hsa05215)	9.959E-05
**Transcriptional misregulation in cancer(hsa05202)**	**1.580E-04**
Endocytosis (hsa04144)	2.308E-04
Oocyte meiosis (hsa04114)	2.410E-04
Regulation of actin cytoskeleton (hsa04810)	3.968E-04
Focal adhesion (hsa04510)	5.100E-04
Thyroid hormone signaling pathway (hsa04919)	6.869E-04
Bacterial invasion of epithelial cells (hsa05100)	9.954E-04
Glutamatergic synapse (hsa04724)	9.954E-04
**PI3K-Akt signaling pathway (hsa04151)**	**1.011E-03**
**Ras signaling pathway (hsa04014)**	**1.515E-03**
Melanoma (hsa05218)	1.708E-03
Glioma (hsa05214)	1.872E-03
GABAergic synapse (hsa04727)	1.959E-03
Arrhythmogenic right ventricular cardiomyopathy (ARVC) (hsa05412)	2.330E-03
Gap junction (hsa04540)	4.817E-03
FoxO signaling pathway (hsa04068)	5.137E-03
Thyroid cancer (hsa05216)	5.369E-03
Inositol phosphate metabolism (hsa00562)	7.351E-03
Acute myeloid leukemia (hsa05221)	8.747E-03
Mucin type O-Glycan biosynthesis (hsa00512)	9.315E-03
Wnt signaling pathway (hsa04310)	9.315E-03
Colorectal cancer (hsa05210)	1.070E-02
Long-term potentiation (hsa04720)	1.070E-02
Adrenergic signaling in cardiomyocytes(hsa04261)	1.285E-02
Oxytocin signaling pathway (hsa04921)	1.620E-02
Amphetamine addiction (hsa05031)	1.954E-02
Shigellosis (hsa05131)	2.067E-02
Long-term depression (hsa04730)	2.067E-02
Dorso-ventral axis formation (hsa04320)	2.232E-02
Phosphatidylinositol signaling system (hsa04070)	2.575E-02
**Ubiquitin mediated proteolysis (hsa04120)**	**2.832E-02**
mRNA surveillance pathway (hsa03015)	3.431E-02
ErbB signaling pathway (hsa04012)	3.431E-02
Mineral absorption (hsa04978)	4.049E-02
**Choline metabolism in cancer (hsa05231)**	**4.363E-02**
Endocrine and other factor-regulated calcium reabsorption (hsa04961)	4.613E-02
Chronic myeloid leukemia (hsa05220)	4.944E-02

### Validation of differential miRNAs

**Figure 3:**
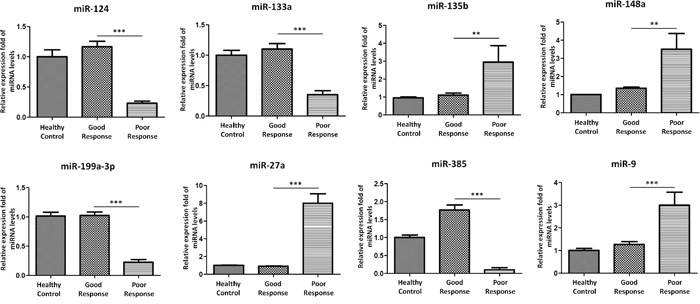
Validation of miRNA array expression using independent samples TaqMan real-time RT-PCR to validate the expression levels of miR-124, miR-133a, miR-135b, miR-148a, miR-199a-3p, miR-27a, miR-385, and miR-9 using an independent cohort of 20OS patients with poor chemotherapeutic response, 20OS patients with good chemotherapeutic response, and 20age, sex matched healthy controls. Data shown are as mean ± SEM.

TaqMan Real-Time PCR was perforrmed to validate the differential microRNA expression levels from microRNA assay. These miRNAs were selected for validation based on the statistic significance and/or biological plausibility. MiR-124, miR-133a, miR-135b, miR-148a, miR-199a-3p, miR-27a, miR-385, and miR-9 were selected for further validation using an independent cohort of 20 OS patients with poor chemotherapeutic response, 20 OS patients with good chemotherapeutic response, and 20 age, sex matched healthy controls (Supplementary Table 2). In agreement with the preliminary data from microRNA assay, miR-124, miR133a, miR-199a-3p and miR-385 were significantly reduced in sera exosomes in poor chemotherapeutic response when compared with good response. While miR-135b, miR-148a, miR-27a, and miR-9 were significantly over expressed in sera exosomes from patients with poor chemotherapeutic response (Figure 3). Taken together, these data confirmed the validity of differentially expressed exosomal miRNAs in OS with poor response to chemotherapy.

### Messenger RNAs were differentially expressed in exosomes and associated with poor response to chemotherapy

**Figure 4:**
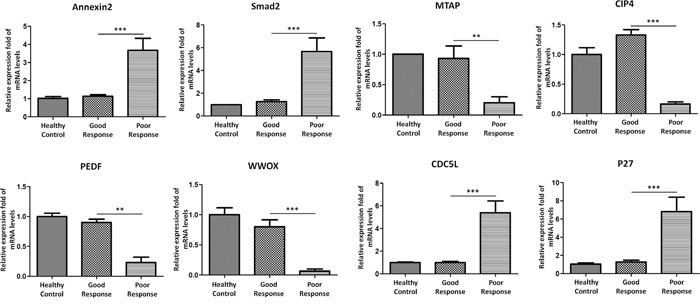
Messenger RNAs were differentially expressed in exosomes in poorly responded patients with OS Annexin2, Smad2, MTAP, CIP4, PEDF, WWOX, Cdc5L, P27 were selected for validation their differential expression in exosomes in independent cohort of 20OS patients with poor chemotherapeutic response, 20OS patients with good chemotherapeutic response, and 20 healthy controls. Data shown are as mean ± SEM.

We further examined the presence of messenger RNAs in exosome and elucidate their roles in poor chemotherapeutic response. We thus employed quantitative RT-PCR to detect the expression of mRNAs isolated from exosome. Eight potential mRNA candidates in osteosarcoma, including Annexin2 [12], Smad2 [13], Methylthioadenosine phosphorylase (MTAP) [14], Cdc42-interacting protein 4 (CIP4) [15], Pigment Epithelium-Derived Factor (PEDF) [16], WW domain-containing oxidoreductase (WWOX) [17], Cell division cycle 5-like (Cdc5L) [18], P27 [19]were selected for quantitative RT-PCR experiments using sera exosomal RNA from 20OS patients with poor chemotherapeutic response, 20OS patients with good chemotherapeutic response, and 20 healthy controls. MTAP, CIP4, PEDF, and WWOX were found to be present and significantly under expressed in sera exosomes of poor chemotherapeutic response when compared with good chemotherapeutic response (Figure 4). However, Annexin2, Smad2, CDC5L, and P27 mRNA levels were over expressed in sera exosomes of poor chemotherapeutic response when compared with good response (Figure 4).

### Evaluation of exosomal miRNAs as the diagnostic biomarkers of poor chemotherapeutic response

**Figure 5:**
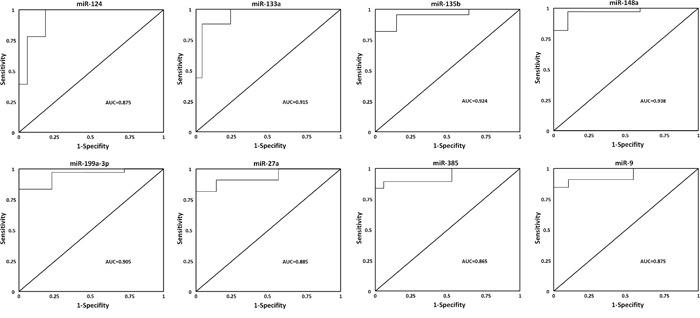
ROC curves for miRNAs that are significantly different in poor chemotherapeutic response as compared to good chemotherapeutic response ROC curve with AUC for miR-124, miR-133a, miR-135b, miR-148a, miR-199a-3p, miR-27a, miR-385, and miR-9 was performed using SPSS.

To evaluate the utility of exosomal miRNAs levels in discriminating OS cases with poor chemotherapeutic response from good response, ROC curve analysis was performed. We confirmed that the eight miRNAs found in the primary analyses highly discriminated OS patients with poor chemotherapeutic response from good response (Figure 5): miR-134, AUC = 0.875, CI_95%_ = (0.822-0.956), miR-133a, AUC = 0.915, CI_95%_ = (0.833-1.067), miR-135b, AUC = 0.924, CI_95%_ = (0.845-1.102), miR-148a (AUC = 0.938, CI_95%_ = (0.843-1.207), miR-199a-3p (AUC = 0.905, CI_95%_ = (0.803-1.017), miR-27a (AUC = 0.885, CI_95%_ = (0.793-0.977), miR-385 (AUC = 0.865, CI_95%_ = (0.773-0.977), and miR-9, AUC = 0.875, CI_95%_ = (0.792-0.963) (p< 0.0001 for all comparisons). Highest area under the curve (AUC) for a single miRNA could be achieved with miR-148a. Further the combination of miR-148a and miR-133acould enhance the performance of discrimination significantly (AUC = 0.999, CI_95%_ = (0.955-1.190)). These data demonstrated that exosomal miRNAs are reliable diagnostic markers for poor chemotherapeutic response in patients with osteosarcoma.

## DISCUSSION

OS remains a devastating disease. It is recently demonstrated that application of microarray technology can yield promising results to classify and diagnose various types of tumors [20, 21]. In this study we have, for the first time, verified the presence of miRNAs and mRNAs in exosomes isolated from sera of OS patients with differential chemotherapeutic response. In addition, a substantial profile of exosomal miRNAs including miR-124, miR-133a, miR-135b, miR-148a, miR-199a-3p, miR-27a, miR-385, and miR-9 was dysregulated in poor chemotherapeutic response. We further demonstrated that exosomal RNAs including Annexin2, Smad2, MTAP, CIP4, PEDF, WWOX, Cdc5L, P27, could also discriminate good and poor chemotherapeutic response for osteosarcoma treatment. These results would assist with potential in clinical chemotherapeutic treatment of OS and help monitoring or predicting disease progression during chemotherapy in osteosarcoma.

MicroRNAs have been demonstrable in the progression of human osteosarcoma. MiR-124 expression was significantly down regulated in osteosarcoma tissues [22]. In addition, miR-124 expression in the metastases OS tissues was significantly lower than non-metastases tissues. Forced expression of miR-124 could suppress Rac1 expression and then attenuated cell proliferation, migration, and invasion *in vitro*. Our results suggest that miR-124 could be a potential target for the chemotherapeutic treatment of osteosarcoma in future. Ji F et al [23] found that miR-133a, reduced in osteosarcoma cells and osteosarcoma tissues, was significantly associated with OS progression. The authors further reported that miR-133a suppressed proliferation and promoted apoptosis by targeting Bcl-xL and Mcl-1. Our current data of miR-133a reduction involved in poor chemotherapeutic treatment in osteosarcoma will provide further evidence on its potential in cancer therapy. Another molecule, miR-135b was frequently up regulated in OS tissues, inversely linked with FOXO1 mRNA expression [24]. In addition, miR-135b knockdown significantly regulated proliferation and invasion. Our data also supported their study, that miR-135b significantly increased in poor chemotherapeutic treatment in osteosarcoma, which provide compelling evidence that miR-135b functions as an onco-miRNA in OS and its oncogenic effects should be candidate target for future OS treatment. MiR-148a expression in sera in OS was significantly higher than normal controls [25]. In addition, they further investigated that miR-148a expression was correlated with tumor size and distant metastasis in OS. Our findings with higher expressed exosomal miR-148a in OS patients with poor response to chemotherapy demonstrated that miR-148a as a novel diagnostic biomarker to identify individuals with poor chemotherapeutic response to OS. Recently, miR-199a-3p was reported to be significantly down regulated in OS [26]. Over expression of miR-199a-3p in osteosarcoma cells can significantly inhibit cell growth and migration. Additionally, they observed mTOR and Stat3 expression was decreased in miR-199a-3p over expressed cells. Our results also demonstrated that exosomal miR-199a-3p significantly lower expressed in poor chemotherapeutic response in osteosarcoma patients, supporting new insights for miR-199a-3p in osteosarcoma chemotherapy and suggesting that miR-199a-3p may play a functional role in treatment of osteosarcoma. Rami Aqeilan's findings [6] of miR-27a and miR-27a* acting as pro-metastatic miRNAs contributed to the idea that star miRNAs are functional in tumor development. Our study for the first time provide evidence that miR-27a functions as an exosomal microRNA in OS cells suggesting that upregulated miR-27a expression with pro-metastatic action in OS. The presented data in our study further suggest the potential of miR-27a as diagnostic markers and therapeutic targets for OS chemotherapy. MiR-9 is considered as a potential up regulated oncogene in OS [27]. MiR-9 knockdown resulted in suppressed colony formation and cell proliferation in OS. These results supported our data that miR-9 was high expressed in poorly chemotherapeutically responded patients, highlighting the importance of miR-9 as an oncogene in osteosarcoma and may provide new insights into chemotherapy of osteosarcoma.

There are some limitations in our study. First, a major limitation of this study is the normalization. U6 should be used with caution, because in some circumstances normalizing by U6 in miRNA expression studies may produce misleading results. Secondly, the multivariate analyses could further explore the differential role of miRNA and mRNA in chemotherapeutic response, such as Fisher discriminant analysis or other statistical methods.

To conclude, our study revealed a substantial abundance of miRNAs and mRNAs present in bloodstream exosome of OS patients. These RNA molecules were differentially expressed in exosome in OS patients, andexosomal miRNAs in seracould be evaluated as reliable diagnostic biomarkers for differential chemotherapeutic response to osteosarcoma. These results provide evidence for the potential value of these biomarkers for the diagnosis and assessment of OS chemotherapy and suggest dysregulation of exosome RNAs abundance for poor chemotherapeutic response to osteosarcoma.

## MATERIALS AND METHODS

### Patients and tumor samples

Written informed consent for participation in the study was obtained from either directly or from his or her guardian in all subjects and the work received approval from the Ethical Committee of Zhejiang Provincial People's Hospital and in accordance with the tenets of the Declaration of Helsinki. The study included a total cohort of 48 OS patients with poor chemotherapeutic response, 45 OS patients with good chemotherapeutic response, and 51 healthy controls for definite diagnosis of osteosarcoma and before receiving preoperative chemotherapy. All tumor samples were classified by two experienced pathologists. The initial biopsy samples were obtained at the time of diagnosis before the initiation of preoperative chemotherapy. The good responders were defined as patients whose tumors had ≥90% necrosis in response to preoperative chemotherapy as determined by histologic examination at the time of definitive surgery and poor responders had <90% necrosis.

### Isolation of exosomes

Exosomes were isolated as described before [28]. Differential centrifugation was performed to isolate exosomes from conditioned medium. Initial spins consisted of a 10-min spin at 1,000 g, a 2,000 g spin for 10 min and a 10,000 g spin for 30 min. The supernatant was retained each time. The supernatant was then spun at 100,000 g for 70 min and the pellet was re-suspended in 1 × PBS, to dilute remaining soluble factors, followed by another centrifugation at 100,000 g for 70 min. The final pellet contained the exosomes, which were re-suspended in tissue culture media.

### RNA processing and miRNA profiling

Exosomal RNA was extracted using the QiagenmiRNeasy Serum/Plasma Kit (Qiagen, Valencia, CA) according to the manufacturer's instructions. RNA quality was assessed by using UV 260/280 and 230/260 absorbance ratios. RNA size distribution was examined on RNA Pico LabChips (Agilent Technologies, Palo Alto, CA) processed on the Bioanalyzer (Agilent). Global miRNA profiling was studied using the TaqMan Low-Density Array Human miRNA Panels (Applied Biosystems, Foster City, CA) containing 746 mature human miRNAs. miRNAs found to be differentially expressed in poor response compared to good response were quantified using TaqMan miRNA assays (Applied Biosystems) modified by the incorporation of our standard curve protocol [29].

### MiRNA target prediction and pathway analysis

Through the use of the bioinformatic tool DIANA-mirPath (v.2) [30]the differentially expressed miRNA were analyzed to predict miRNA targets in 3’-UTR gene regions according to experimentally validated miRNA interactions derived from DIANA-TarBase v6.0 algorithm. These interactions (predicted and/or validated) were subsequently combined with sophisticated merging and meta-analysis algorithms by DIANA-mirPath.

### TaqMan miRNA assay for individual miRNAs

Real Time PCR analysis was performed on the ABI Prism 7000 Sequence Detection System (Applied Biosystems) using 3 μl of RT products in a reaction mixture containing TaqMan miRNA assay and the TaqMan Universal PCR Master Mix, according to the manufacturer's instructions (Applied Biosystems). All PCR reactions were performed in triplicate including no-template controls. Relative quantities of each miRNA were calculated using the ΔΔCt method after normalization with endogenous reference U6.

### Quantitative real-time PCR (Sybr green QPCR)

Total RNA were reverse transcribed in 20 μl using the High Capacity cDNA Reverse Transcription Kit, according to the manufacturer's protocol (Applied Biosystem). Quantitative real-time PCR was performed using 80 ng of cDNA in a final volume of 20 μl according to the manufacturer's instructions (Applied Biosystems), on the ABI Prism 7000 Detection System (Applied Biosystems). Relative expression was calculated using the comparative Ct method. All PCRs were performed in triplicate including no-template controls.

### Statistical analysis

Data analysis on miRNA expression levels from TaqMan Low-Density Array was performed by SDS software version 2.2.2 (Applied Biosystems). The delta Ct values were calculated by using U6 as the endogenous controls. Heatmap of differentially expressed miRNAs and between-group statistic analysis were performed by R software. A receiver operating characteristic (ROC) curve was performed to calculate the relationship between sensitivity and specificity for disease group versus healthy controls. Data were analysed using Student's t-tests, to determine statistically significant differences between relevant samples. P-values were either listed or represented by the following number of asterisks: *P<0.05; **P<0.01; ***P<0.001; ****P<0.0001.

## SUPPLEMENTARY MATERIALS FIGURES AND TABLES



## References

[1] Miller RW (1981). Contrasting epidemiology of childhood osteosarcoma, Ewing's tumor, and rhabdomyosarcoma. Natl Cancer Inst Monogr.

[2] Ottaviani G, Jaffe N (2009). The epidemiology of osteosarcoma. Cancer Treat Res.

[3] Provisor AJ, Ettinger LJ, Nachman JB, Krailo MD, Makley JT, Yunis EJ, Huvos AG, Betcher DL, Baum ES, Kisker CT, Miser JS (1997). Treatment of nonmetastatic osteosarcoma of the extremity with preoperative and postoperative chemotherapy: a report from the Children's Cancer Group. J Clin Oncol.

[4] Bartel DP (2004). MicroRNAs: genomics, biogenesis, mechanism, and function. Cell.

[5] Jones KB, Salah Z, Del Mare S, Galasso M, Gaudio E, Nuovo GJ, Lovat F, LeBlanc K, Palatini J, Randall RL, Volinia S, Stein GS, Croce CM (2012). miRNA signatures associate with pathogenesis and progression of osteosarcoma. Cancer Res.

[6] Salah Z, Arafeh R, Maximov V, Galasso M, Khawaled S, Abou-Sharieha S, Volinia S, Jones KB, Croce CM, Aqeilan RI (2015). miR-27a and miR-27a* contribute to metastatic properties of osteosarcoma cells. Oncotarget.

[7] Lässer C, Alikhani VS, Ekström K, Eldh M, Paredes PT, Bossios A, Sjöstrand M, Gabrielsson S, Lötvall J, Valadi H (2011). Human saliva, plasma and breast milk exosomes contain RNA: uptake by macrophages. J Transl Med.

[8] Valadi H, Ekström K, Bossios A, Sjöstrand M, Lee JJ, Lötvall JO (2007). Exosome-mediated transfer of mRNAs and microRNAs is a novel mechanism of genetic exchange between cells. Nat Cell Biol.

[9] Yuan A, Farber EL, Rapoport AL, Tejada D, Deniskin R, Akhmedov NB, Farber DB (2009). Transfer of microRNAs by embryonic stem cell microvesicles. PLoS One.

[10] Aucher A, Rudnicka D, Davis DM (2013). MicroRNAs transfer from human macrophages to hepato-carcinoma cells and inhibit proliferation. J Immunol.

[11] Xu JF, Yang GH, Pan XH, Zhang SJ, Zhao C, Qiu BS, Gu HF, Hong JF, Cao L, Chen Y, Xia B, Bi Q, Wang YP (2014). Altered microRNA expression profile in exosomes during osteogenic differentiation of human bone marrow-derived mesenchymal stem cells. PLoS One.

[12] Gillette JM, Chan DC, Nielsen-Preiss SM (2004). Annexin 2 expression is reduced in human osteosarcoma metastases. J Cell Biochem.

[13] Won KY, Kim YW, Park YK (2010). Expression of Smad and its signalling cascade in osteosarcoma. Pathology.

[14] Miyazaki S, Nishioka J, Shiraishi T, Matsumine A, Uchida A, Nobori T (2007). Methylthioadenosine phosphorylase deficiency in Japanese osteosarcoma patients. Int J Oncol.

[15] Koshkina NV, Yang G, Kleinerman ES (2013). Inhibition of Cdc42-interacting protein 4 (CIP4) impairs osteosarcoma tumor progression. Curr Cancer Drug Targets.

[16] Broadhead ML, Dass CR, Choong PF (2011). Systemically administered PEDF against primary and secondary tumours in a clinically relevant osteosarcoma model. Br J Cancer.

[17] Zhang N, Jiang Z, Ren W, Yuan L, Zhu Y (2016). Association of polymorphisms in WWOX gene with risk and outcome of osteosarcoma in a sample of the young Chinese population. Onco Targets Ther.

[18] Martin JW, Chilton-MacNeill S, Koti M, van Wijnen AJ, Squire JA, Zielenska M (2014). Digital expression profiling identifies RUNX2, CDC5L, MDM2, RECQL4, and CDK4 as potential predictive biomarkers for neo-adjuvant chemotherapy response in paediatric osteosarcoma. PLoS One.

[19] Li Y, Nakka M, Kelly AJ, Lau CC, Krailo M, Barkauskas DA, Hicks JM, Man TK (2016). p27 Is a Candidate Prognostic Biomarker and Metastatic Promoter in Osteosarcoma. Cancer Res.

[20] Ramaswamy S, Golub TR (2002). DNA microarrays in clinical oncology. J Clin Oncol.

[21] Liu ET (2003). Classification of cancers by expression profiling. Curr Opin Genet Dev.

[22] Geng S, Zhang X, Chen J, Liu X, Zhang H, Xu X, Ma Y, Li B, Zhang Y, Bi Z, Yang C (2014). The tumor suppressor role of miR-124 in osteosarcoma. PLoS One.

[23] Ji F, Zhang H, Wang Y, Li M, Xu W, Kang Y, Wang Z, Wang Z, Cheng P, Tong D, Li C, Tang H (2013). MicroRNA-133a, downregulated in osteosarcoma, suppresses proliferation and promotes apoptosis by targeting Bcl-xL and Mcl-1. Bone.

[24] Pei H, Jin Z, Chen S, Sun X, Yu J, Guo W (2015). MiR-135b promotes proliferation and invasion of osteosarcoma cells via targeting FOXO1. Mol Cell Biochem.

[25] Ma W, Zhang X, Chai J, Chen P, Ren P, Gong M (2014). Circulating miR-148a is a significant diagnostic and prognostic biomarker for patients with osteosarcoma. Tumour Biol.

[26] Duan Z, Choy E, Harmon D, Liu X, Susa M, Mankin H, Hornicek F (2011). MicroRNA-199a-3p is downregulated in human osteosarcoma and regulates cell proliferation and migration. Mol Cancer Ther.

[27] Zhu SW, Li JP, Ma XL, Ma JX, Yang Y, Chen Y, Liu W (2015). miR-9 Modulates Osteosarcoma Cell Growth by Targeting the GCIP Tumor Suppressor. Asian Pac J Cancer Prev.

[28] Jansen FH, Krijgsveld J, van Rijswijk A, van den Bemd GJ, van den Berg MS, van Weerden WM, Willemsen R, Dekker LJ, Luider TM, Jenster G (2009). Exosomal secretion of cytoplasmic prostate cancer xenograft-derived proteins. Mol Cell Proteomics.

[29] Anglicheau D, Sharma VK, Ding R, Hummel A, Snopkowski C, Dadhania D, Seshan SV, Suthanthiran M (2009). MicroRNA expression profiles predictive of human renal allograft status. Proc Natl Acad Sci USA.

[30] Papadopoulos GL, Alexiou P, Maragkakis M, Reczko M, Hatzigeorgiou AG (2009). DIANA-mirPath: integrating human and mouse microRNAs in pathways. Bioinformatics.

