# Supplementary material for: Exosomes containing differential expression of microRNA and mRNA in osteosarcoma that can predict response to chemotherapy
Source: Oncotarget. 2017 Jun 6;8(44):75968–78. doi: 10.18632/oncotarget.18373 (PMC5652678; doi:10.18632/oncotarget.18373)
Supplement: Supplementary file 1 [file oncotarget-08-75968-s001.pdf]

Exosomes containing differential expression of microRNA and mRNA in osteosarcoma that can predict response to chemotherapy

SUPPLEMENTARY MATERIALS

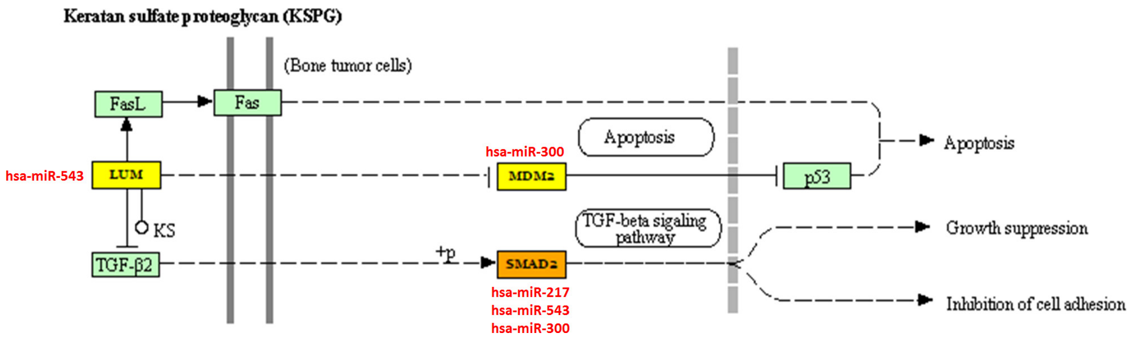

Supplementary Figure 1: The KEGG pathway Proteoglycans in cancer was significantly altered in poor chemotherapeutic response.

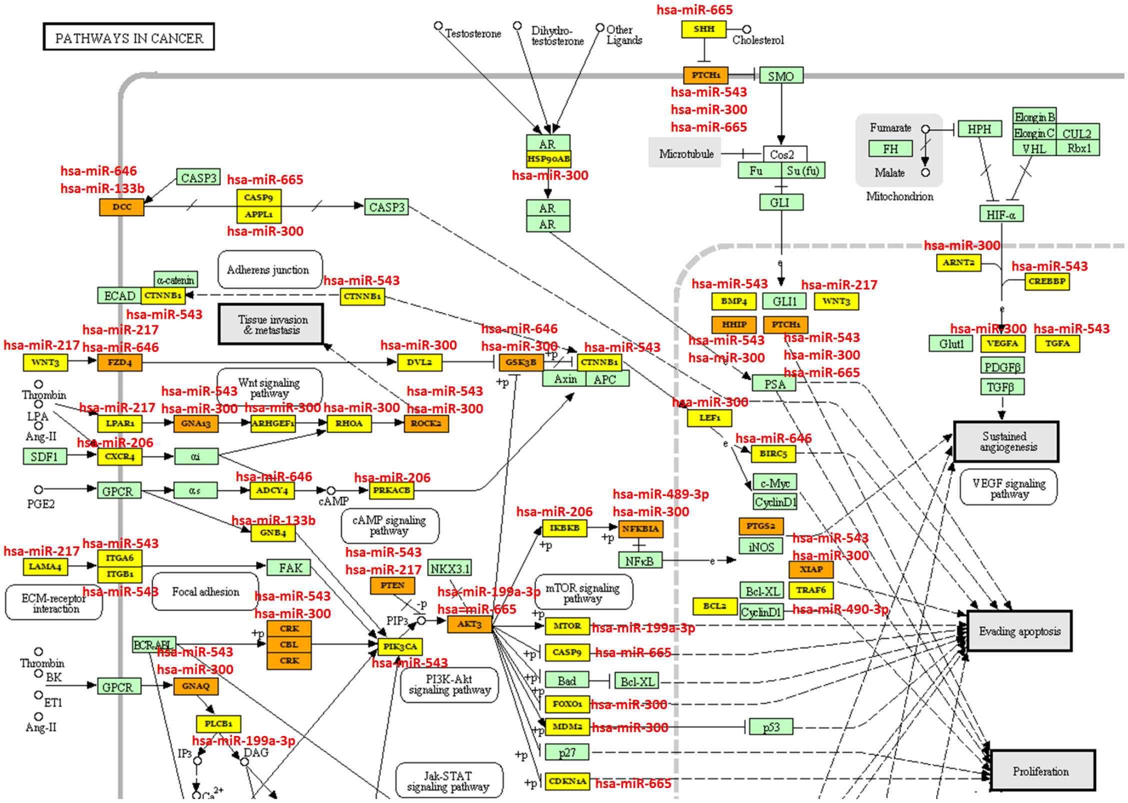

Supplementary Figure 2: The KEGG pathway Pathways in cancer was also significantly altered in poor chemotherapeutic response.

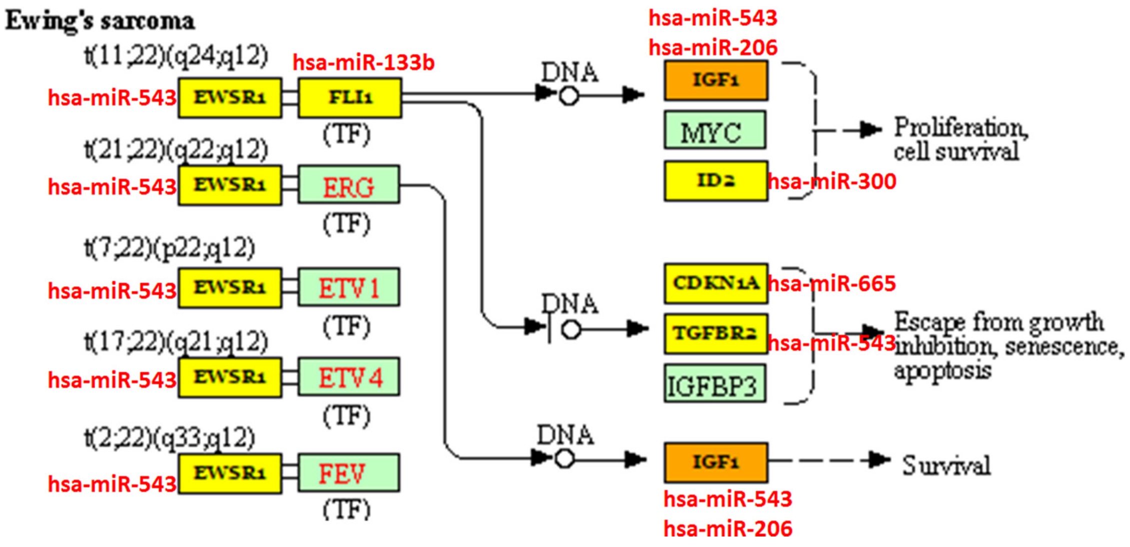

Supplementary Figure 3: The KEGG pathway Transcriptional misregulation in cancer was significantly enriched in poor chemotherapeutic response.

Supplementary Table 1: Clinical information of the OS sample used in pilot study

Clinical features	Healthy control	OS with good response	OS with poor response
Patient Number	31	25	28
Gender (f/m)	18/13	15/10	18/10
Age (y)	18±7	17±9	17±10
Metastatic status	nonmetastatic	nonmetastatic	15 metastatic
Hisologic response	good response	good response	poor response
Recurrence	NA	2 recurrence	22 recurrence
Follow-up (mo)	NA	36±18	48±22

Supplementary Table 2: Clinical information of the OS sample used in validation cohorts

Clinical features	Healthy control	OS with good response	OS with poor response
Patient Number	20	20	20
Gender (f/m)	10/10	10/10	10/10
Age (y)	17±8	18±10	19±7
Metastatic status	nonmetastatic	nonmetastatic	17 metastatic
Hisologic response	good response	good response	poor response
Recurrence	NA	1 recurrence	15 recurrence
Follow-up (mo)	NA	35±13	55±23
